# Medical and Physical Intelligence

**Published:** 1804-08-01

**Authors:** 


### MEDICAL AND PHYSICAL INTELLIGENCE.

[ FOREIGN AND DOMESTIC. ]

### *Mr. Buchholz's new Method of preparing Emetic Tartar.*

Take of crystals of tartar, two pounds; glass of antimony, one pound and a half, let them be well pulverised, and mix them together. Then pour on the mixture a sufficient quantity of water to make a thick paste. Expose this mixture under a glass bell to the action of the solar rays for about a fortnight, during which time it must be agitated three or four times a day, and a little water added to replace that which evaporates, in order to keep the mass in the same consistency. The mass puffs up, and sulphurated hydrogen gas is disengaged. Flakes of an analogous colour to kermes are perceived, and the whole mass receives a red brown colour. At the end of a fortnight, it is dissolved and washed with boling water, and afterwards filtrated. The insoluble residuum amounts to about three ounces. On evaporating the liquor, two pounds and fifteen ounces of emetic tartar in fine crystals will be obtained. From Mr. B's experiments with the residuum and the crystals of emetic tartar, he concluded,

1. That the glass of antimony may be dissolved by the acidulous tartrit of pot-ash in the state of a paste, and at a middle temperature.

2 That it is only necessary to repeat the crystallization, in order to deprive emetic tartar of iron, and of tartrit of lime.

3. That silica is not found in all glasses of antimony.

4. That under certain circumstances, particularly through the assistance of other saline substances, the tartrit of lime dissolves in great quantity, and even crystallizes regularly.

5. That one part of crystallized emetic tartar requires about fourteen parts of distilled water of 10—12° Reaumur for solution, and not eighty parts, as some chemists have imagined.

6. That 100 parts of boiling water are capable of dissolving 53 parts of emetic tartar, when entirely free from tartrit of iron and tartrit of lime.

*BILL*

### *BILL OF MORTALITY,*

### For Portsmouth, New Hampshire, for A. D. 1802.

By LYMAN SPALDING, M.B. &c.

| COMPLAINT. | AGE. | Jan. | Feb. | Mar. | Apr. | May | June | July | Aug. | Sept. | Oct. | Nov. | Dec. | Total. |
|---|---|---|---|---|---|---|---|---|---|---|---|---|---|---|
| Aphtha – – – – – – – – – – – | 4, 4 weeks | | | | | | | | | 2 | | | | 2 |
| Apoplexy – – – – – – – – | 66, 33, 55, 43, 63 years | 1 | | 1 | | | | | 1 | | 1 | 1 | | 5 |
| Atrophy – – – – – – | 55, 69, 40, 55, 3m. 60 years | | | 1 | | | | 1 | | 1 | | | 3 | 6 |
| Cancer – – – – – – – – – – – | 55, 63, 60 years | | | | | | | | | | 1 | 2 | | 3 |
| Cankerrash – – – – | 8, 2, 5, 7m. 2, 16, 23, 4 years | | | | | | | | | | 1 | 1 | 6 | 8 |
| Cholera of Infants – – – – – – – | 6 to 24 months | | | | | | | 1 | 6 | 2 | 3 | | 1 | 13 |
| Cholic bilious – – – – – – – – – – | 42 years | | | | | | | | | | | 1 | | 1 |
| Consumption | 14, 74, 53, 47, 53, 30, 69, 17, 33, 30, 60, 14, 33, 18, 69, 64, 60, 33, 28, 48, 52, 18, 29, 50, 63, 28, 22, 30 – – – – – – | 2 | 2 | 4 | 3 | 7 | | 1 | 3 | | 2 | 2 | 2 | 28 |
| Debauchery – – – – – – – – – – – | 55, 38 years | | | | | | 1 | | | | | 1 | | 2 |
| Dropsy – – – – – – – | 69, 50, 84, 52, 89, 24 years | | 1 | | | 1 | | 1 | | 2 | | | 1 | 6 |
| Dropsy in the brain – – – – – – | 3, 7, 7, 8, 13 years | 1 | 1 | 1 | | | 1 | | 1 | | | | | 5 |
| Dysentery – – – – – – – – – – | 3, 2, 2 years | | | | | | | | | 2 | 1 | | | 3 |
| Epilepsy – – – – – – – – – – | 64, 2, 2 years | | | | | | | | | 1 | 2 | | | 3 |
| Fever and Ague – – – – – – – – – – | 33 years | | | | | | | | | | 1 | | | 1 |
| Fever bilious – – – – – – – – – | 74, 30, 27 years | | | 1 | 1 | | | | | | | 1 | | 3 |
| Fever bilious malignant | 44, 31, 41, 13, 35, 21, 30, 40, 30, 13 – – – – – – | | | | | | | 1 | 9 | | | 1 | | 10 |

| | | | | | | | | | | | | | |
|---|---|---|---|---|---|---|---|---|---|---|---|---|---|
| Fever pulmonic | 65, 45 years | 2 | | | | | | | | | | | 2 |
| Gout | 52 years | | | | | 1 | | | | | | | 1 |
| Gravel | 41 years | | | | | | | | 1 | | | | 1 |
| Hooping cough | 10 weeks to 1 year | 3 | 3 | | | | 1 | | | 1 | | | 8 |
| Infantile complaints | 6 days 4 weeks | | | 1 | | | | 1 | | | | | 2 |
| Measles | 7, 1, 20, 4, 9m. 2, 7, 1, 2, 1, 2 years | | | | 1 | 4 | 5 | 1 | | | | | 11 |
| Mortification | 7 m. 1 year | | | 1 | | | | | | 1 | | | 2 |
| Old age | 94, 90, 78, 76 years | | | 2 | | | | | | | | 2 | 4 |
| Palsy | 60, 74, 64, 50 years | 1 | 1 | | | | | | 1 | | | 1 | 4 |
| Phrenitis | 30, 12 years | | | | | 1 | | | 1 | | | | 2 |
| Premature birth | | 2 | | | 1 | 1 | 1 | | | | 1 | | 6 |
| Quinsy | 3 years | | | | | 1 | | | | | | | 1 |
| Scald head | 1 year | | 1 | | | | | | | | | | 1 |
| Small pox, natural | 33 years | | | 1 | | | | | | | | | 1 |
| Small pox, inoculated | 1 year | | | 1 | | | | | | | | | 1 |
| *Casualties.* Drowned | 48, 60 years | | | | | 1 | | | | | | 1 | 2 |
| *Casualties.* Fall | 55 years | | | | | | | | | | | 1 | 1 |
| *Casualties.* Frozen | 82 years | | | | | | | | | | | | 1 | 1 |
| *Casualties.* Poisoned by opium | 4 months | | | | | | | | 1 | | | | 1 |
| *Casualties.* Suicide | 32 years | | 1 | | | | | | | | | | 1 |
| | | | | | | | | | | | | Total | 152 |

The town has been very unhealthful, some epidemic having raged the whole year. The hooping cough in January and February was very prevalent, and some sporadic cases continued till September. The measles made its appearance about the middle of March, and was very prevalent till July; at which time a bilious malignant fever made its appearance, and continued till August; when the cholera and cankerrash commenced, and continued through the year.

Mr. SCHMIDT prepares ammoniated iron by dissolving muriat of ammonia in two ounces of water, and mixing with it one drachm of muriat of iron in a deliquescent state, which mixture is then evaporated to dryness.

---

Mr. J. HEATH, of Grocer's Hall Court, has lately given an instance of a young gentleman in the last stage of typhus fever, being cured by the use of yeast.

---

Dr. PATTERSON, of Londonderry, will speedily publish, OBSERVATIONS on the Climate of Ireland, and RESEARCHES concerning its Nature, from very early periods to the present Time. With THOUGHTS on some Branches of Rural Economy, particularly recommended in An ADDRESS to the Inhabitants and Friends of this Country. To which are prefixed, PRELIMINARY CONSIDERATIONS on the Structure and Functions of Plants—on the Analogy between the Vegetable and Animal System—on the general State of Woods and Plantations, in Ireland, in ancient and modern Times—on peculiar circumstances denoting the various conditions of its Linen Manufacture throughout a series of ages—and on the Utility of a co-operation in works of Art and Science.

This is a *new* work; and is the *first* undertaken in Ireland, to give a connected and concordant view of subjects manifestly of prime importance to the nation. For what can be estimated more valuable to the people of this fertile and favoured isle, than an union of the principles of science with the rules of art, to raise the bounties of Nature to their highest possible degree of perfection, by a skilful and diligent culture of the necessaries of life—those for food, raiment, and dwelling?

---

CUVIER, the well known natural historian, has lately published a work on the species of animals that have been lost.

---

COURET DE VILLENEUVE has lately published a botanical work at Paris, containing a description of all the plants that are cultivated in the garden of the Central College at Ghent, arranged according to the Linnean system.

